# Comparative Changes in Tissue Oxygenation Between Laparoscopic and Open Cholecystectomy

**DOI:** 10.14740/jocmr2086w

**Published:** 2015-02-09

**Authors:** George D. Bablekos, Stylianos A. Michaelides, Antonis Analitis, Maria H. Lymperi, Konstantinos A. Charalabopoulos

**Affiliations:** a Department of Experimental Physiology, Medical School, Democritous University of Thrace, 68100 Dragana, Alexandroupolis, Greece; b Technological Educational Institute (T.E.I.) of Athens, Agiou Spyridonos 28 Street, 12243 Egaleo, Athens, Greece; c “Sismanogleio” General Hospital, First Thoracic Medicine Department, Sismanogleiou 1 Street, 15126 Maroussi, Athens, Greece; d Department of Hygiene, Epidemiology and Medical Statistics, Medical School, National and Kapodistrian University of Athens, Mikras Asias 75 Street, 11527 Goudi, Athens, Greece; e Department of Experimental Physiology, Medical School, National and Kapodistrian University of Athens, Mikras Asias 75 Street, 11527 Goudi, Athens, Greece

**Keywords:** Laparoscopic, Open, Cholecystectomy, Arterial oxygenation

## Abstract

**Background:**

Previous studies examined the effect of laparoscopic cholecystectomy (LC) versus open cholecystectomy (OC) on physiological variables of the respiratory system. In this study we compared changes in arterial blood gases-related parameters between LC and OC to assess their comparative influence on gas exchange.

**Methods:**

We studied 28 patients, operated under identical anesthetic protocol (LC: 18 patients, OC: 10 patients). Measurements were made on the morning before surgery (BS), the second (AS2) and the eighth (AS8) postoperative day. Studied parameters, including alveolar-arterial difference in PO_2_ ((A-a)DO_2_) and oxygen content (Oct in vol%), were statistically compared.

**Results:**

On AS2 a greater increase was found in ((A-a)DO_2_) for the OC compared to LC (4.673 ± 0.966 kPa versus 3.773 ± 1.357 kPa, respectively). Between BS and AS2, Oct in vol% decreased from 17.55 ± 1.90 to 15.69 ± 1.88 in the LC and from 16.99 ± 2.37 to 14.62 ± 2.23 in the OC, whilst a reduction (P = 0.093) between AS2 and AS8 was also found for the open method. Besides, on AS2, SaO_2_% decrease was greater in OC compared to LC (P = 0.096).

**Conclusions:**

On AS2, the greater increase in OC-((A-a)DO_2_) associated with Oct in vol% and SaO_2_% findings also in OC group suggest that LC might be associated with lower risk for impaired tissue oxygenation.

## Introduction

Laparoscopic cholecystectomy (LC) was performed for the first time in Lyon, France, by Philip Mouret in 1987 [1]. At that time, the influence of conventional upper abdominal surgery on respiratory performance had already been reported in literature [2-5]. An increase of postoperative pulmonary complications associated with altered values in pulmonary function variables had been observed [2-5]. From 1990 to date, LC and open cholecystectomy (OC) have been compared between them in a number of studies [6-25]. Pulmonary function tests, arterial blood gases (ABGs)-related parameters and control of breathing indices values, were all examined before and after both surgical methods in different across time postoperative measurements [6-25]. The studied ABGs-related parameters for either LC or OC were pH, the arterial blood partial pressures PaO_2_ and PaCO_2_, the alveolar-arterial difference in PO_2_ ((A-a)DO_2_), the arterial blood oxygen saturation (SaO_2_%) and the (HCO_3_^-^) concentration. According to literature, the aforementioned ABGs-related variables were specifically measured preoperatively and in different across time instants during the postoperative period, ranging from the time of the patient’s entry into the operating theater up to the eighth postoperative day [10-12, 14-18, 21-25].

To our knowledge, although since 1990 LC has been widely introduced into clinical practice and a great number of studies supported its superiority versus the OC, changes of oxygen content (Oct in vol%) and ((A-a)DO_2_) [14] parameters values, have not either been reported or studied [14] for a time period longer than the first 24 h after surgery, respectively. In our previous works [18, 20] changes of lung volumes, flow rates and control of breathing indices have in detail been analyzed and discussed.

The present study adds information on changes in oxygenation-related variables between LC and OC methods. The aims of our article are summed up in the following clinical research questions: 1) to make a concentrated overall presentation of what has been published concerning changes of ABGs-related parameters after LC and OC, from January 1, 1990 to present; 2) to investigate the changes of ((A-a)DO_2_) and Oct in vol% variables reflecting pulmonary gas exchange and tissue oxygenation respectively, for a relatively extended period after surgery; 3) to discuss and compare the findings of our investigation with those of the relevant literature by further clarifying the effects on oxygenation of upper abdominal surgery.

This might be particularly useful for patients whose respiratory integrity is not considered functionally ideal.

## Patients and Methods

### Patients

The data of the study arise from an experimental work, approved from the Department of Experimental Physiology, Medical School, National and Kapodistrian University of Athens and the Research Ethics Committee of “Korgialeneio-Benakeio” Red Cross General Hospital, Athens, Greece, where the experimental work has taken place. Different parts of this experimental work have previously been reported in literature [18, 20]. A written consent was obtained from all participants, which should have had only symptomatic cholecystolithiasis without cystic or common bile duct gallstones. A complete preoperative screening, including chest radiographs, standard blood and biochemistry tests, cardiac and upper abdominal medical examinations (electrocardiogram, upper abdominal ultrasonography), was performed before surgery for all patients [18, 20]. All were devoid of any other signs and/or symptoms related to cardiopulmonary, metabolic, oncologic, neurologic or immunologic disorders, and were operated in “Korgialeneio-Benakeio” Red Cross General Hospital of Athens for their gallbladder’s lithiasis. The absence of either any type of systematic medical treatment or previous abdominal and/or thoracic surgical operation was also set up as a prerequisite for participants in order to be enrolled in the study.

Participants’ enrollment was brought off by the principal investigator (George D. Bablekos). The day following admission to hospital, after patient’s registration accomplishment at the Emergency Department by the surgical resident on call, the random allocation (no blocking and no stratification) was performed. This was based on patients’ registration at the Emergency Department, by creating triads, and it was conducted for the whole allocation period by the principal investigator as well. In each triad, the first patient was for the OC and the following two patients for the LC surgical procedure. The random allocation was kept confidential until the assignment of surgical operations. Fifty-three (53) patients were assessed for eligibility, out of which 32 were randomly allocated and finally 28 patients (20 female and eight male; LC group: 18 patients, OC group: 10 patients) [18, 20] were included in the study. Mean ± SD values for age and weight of participants were found to be 52.56 ± 12.16 years and 72.22 ± 10.90 kg for the LC group, and 54.8 ± 9.21 years and 79.10 ± 10.04 kg for the OC group respectively [18, 20].

Moreover, nine participants of the 28 (five in the LC and four in the OC group) smoked less than 10 cigarettes per day [18, 20].

### Medical drugs and chemicals administered throughout the surgical operation

The anesthetic protocol was the same for both the LC and the OC groups [18, 20]. Patients’ preparation [18, 20], 1 h before induction of general anesthesia and surgery, was performed via oral administration of 150 mg of ranitidine hydrochloride, while at the same time period 0.5 mg/kg meperidine hydrochloride and cephamandol nafate 1 g/vial × 2 were both intramuscularly administered as well. General anesthesia was administered intravenously (fentanyl citrate, 2 μg/kg; propofol, 2 mg/kg; atracurium besylate, 0.5 mg/kg) [18, 20] for all patients. Maintenance [18, 20] of general anesthesia was ensured through a mixture of 4 L O_2_, 4 L N_2_O plus 1% desflurane at an end-tidal concentration of 6.1% (1 MAC, i.e. 1 minimum alveolar concentration). Atracurium and fentanyl were continuously administered throughout the surgical procedure in both groups [18, 20]. Ten minutes prior to the termination of the operation, atracurium and desflurane were both simultaneously interrupted [18, 20]. Also, neostigmine methylsulfate (0.5 mg/kg) and atropine sulfate (0.02 mg/kg) were used to reverse neuromuscular blockade immediately at the minimal sings of muscular function [18, 20]. Moreover, OC was performed by a 15 cm right oblique sub-costal incision, Kocher incision, and the LC procedure by a standard four-trocar technique (the surgeon on the left of the patient) [18, 20]. The duration for the open procedure (OC) ranged from 80 to 100 min and for the laparoscopic one (LC) from 60 to 80 min [18, 20].

### Measurements

Arterial blood samples for all participants were obtained from the radial artery in the sitting position on breathing room air as follows: in the morning prior to surgery, 2 days after surgery (when patients were still hospitalized) and on the eighth postoperative day (when all had been already discharged from hospital). Analgesics were not administered in any preoperative and/or postoperative measurements, and for all participants. The automatic ABL 3 Radiometer Copenhagen analyzer Denmark was used, and the recorded parameters were: PaO_2_ in mm Hg, PaCO_2_ in mm Hg, pH, (HCO_3_^-^) concentration (mEq/L), the arterial’s blood oxygen content (Oct in vol%), and the arterial’s blood oxygen saturation (SaO_2_%). The alveolar-arterial difference in PO_2_ ((A-a)DO_2_) was calculated in mm Hg using the alveolar-ventilation equation [26].

The mean ± SD values for pH, PaO_2_ and PaCO_2_ in mm Hg have merely numerically reported [18] without the percentage (%) changes and statistical comparisons for their changes either across time per technique or between the two methods, but have also not been analyzed or discussed. Besides, the mean ± SD values for pH [18], PaO_2_ [18] and PaCO_2_ [18] are used in the present manuscript to support their medical interpretation. Also, the mean ± SD in mm Hg values for PaO_2_ [18], PaCO_2_ [18] and the ((A-a)DO_2_) difference were all converted and for all measurements to kPa (1 mm Hg = 0.133 kPa exactly).

The recorded across time measurements were performed and compared for each surgical group preoperatively versus the second and the eighth postoperative day. Moreover, for both groups, mean percentage (%) changes from baseline versus the second and the eighth postoperative day were also calculated.

### Statistics

Comparisons of preoperative values between LC and OC surgical groups were performed by using the *t*-test or the Welch test in case of unequal variances. The across time changes for each group (preoperative compared to the second and eighth postoperative day values) were determined by using the one-factor repeated measures ANOVA. Statistically significant results were followed by pairwise multiple comparisons procedure using Bonferroni correction.

To evaluate across time differences between LC and OC groups, mean percentage changes and 95% confidence intervals (CIs) from preoperative versus the second and eighth postoperative days were firstly calculated for all the examined parameters. In either surgical group and for each variable, mean percentage changes were calculated by determining the percentage change for each patient separately from preoperative versus the second and the eighth postoperative day values and then calculating the mean for the second and eighth postoperative days for each group. Comparisons between the LC and OC groups were performed by using the *t*-test or the Welch test as appropriate. All tests were two-sided, and the level of significance was set at P = 0.05. For all analyses the SPSS for windows version 18.00 was used.

## Results

Numerical values concerning the across time per technique measurements along with their mean percentage changes (%) and 95% CIs for all the examined and medically interpretated variables, are presented in Table 1 for the LC and in Table 2 for the OC group.

**Table 1:** Studied ABGs-Related Parameters for the Laparoscopic Cholecystectomy Group

Studied parameters	Preoperative value mean ± SD	Second postoperative day value mean ± SD	Changes from preoperative to second postoperative day mean ± SEM (95% CI), %	Eighth postoperative day value mean ± SD	Changes from preoperative to eighth postoperative day mean ± SEM (95% CI), %
(A-a)DO_2_, kPa (mm Hg)	3.428 ± 1.458 (25.71 ± 10.94)	3.773 ± 1.357 (28.30 ± 10.18)	44.0493 ± 32.6119 (-26.404, 114.503)	2.781 ± 1.296 (20.86 ± 9.72)	4.3974 ± 22.1251 (-43.401, 52.196)
HCO_3_^-^, mEq/L	24.14 ± 1.10	25.09 ± 2.11	6.445 ± 0.697 (1.30, 11.59)	24.47 ± 1.45	3.474 ± 1.001 (-3.22, 10.17)
SaO_2_%	94.93 ± 2.11	94.93 ± 1.83	-0.2357 ± 0.2087 (-1.778, 1.307)	95.82 ± 2.47	1.097 ± 0.5786 (-2.773, 4.967)
Oct in vol%	17.55 ± 1.90	15.69 ± 1.88	-10.71 ± 1.34 (-20.62, -0.80)	15.98 ± 1.73	-7.095 ± 1.632 (-18.01, 3.82)
pH	7.41 ± 0.03	7.43 ± 0.03	0.2747 ± 0.1072 (0.044, 0.507)	7.43 ± 0.03	0.1825 ± 0.1348 (-0.109, 0.474)
PaO_2_, kPa (mm Hg)	10.521 ± 2.322 (78.91 ± 17.42)	9.988 ± 1.586 (74.71 ± 11.90)	- 3.6965 ± 3.4823 (-11.219, 3.827)	10.96 ± 1.492 (82.20 ± 11.19)	7.7561 ± 6.4765 (-6.236, 21.748)
PaCO_2_, kPa (mm Hg)	5.112 ± 0.308 (38.34 ± 2.31)	4.984 ± 0.413 (37.38 ± 3.10)	- 2.3400 ± 2.2046 (-7.103, 2.423)	4.94 ± 0.370 (37.05 ± 2.78)	- 3.1716 ± 2.0601 (-7.622, 1.279)

*Values in pH, PaO_2_ mm Hg and PaCO_2_ mm Hg have been numerically reported in a previous work [18].

**Table 2:** Studied ABGs-Related Parameters for the Open Cholecystectomy Group

Studied parameters	Preoperative value mean ± SD	Second postoperative day value mean ± SD	Changes from preoperative to second postoperative day mean ± SEM (95% CI), %	Eighth postoperative day value mean ± SD	Changes from preoperative to eighth postoperative day mean ± SEM (95% CI), %
(A-a)DO_2_, kPa (mm Hg)	3.776 ± 1.949 (28.32 ± 14.62)	4.673 ± 0.966 (35.05 ± 7.25)	148.1581 ± 123.4195 (-131.04, 427.35)	2.857 ± 1.556 (21.43 ± 11.67)	17.5287 ± 36.7968 (-65.71, 100.77)
HCO_3_^-^, mEq/L	24.19 ± 3.97	25.71 ± 1.72	8.159 ± 3.151 (-12.92, 29.23)	25.01 ± 1.19	8.540 ± 3.343 (-13.10, 30.18)
SaO_2_%	95.34 ± 2.19	93.6 ± 1.41	-1.8307 ± 0.1626 (-2.918, -0.7433)	94.99 ± 2.66	-0.2117 ± 0.5607 (-3.8415, 3.4179)
Oct in vol%	16.99 ± 2.37	14.62 ± 2.23	-11.31 ± 2.318 (-26.81, 4.19)	16.10 ± 2.38	0.248 ± 3.109 (-19.88, 20.37)
pH	7.44 ± 0.02	7.44 ± 0.03	0.07508 ± 0.1091 (-0.171, 0.323)	7.42 ± 0.03	-0.1938 ± 0.1207 (-0.466, 0.080)
PaO_2_, kPa (mm Hg)	10.244 ± 2.025 (76.83 ± 15.19)	9.086 ± 1.004 (68.15 ± 7.53)	- 8.9566 ± 5.4844 (-21.363, 3.450)	10.5 ± 1.617 (78.75 ± 12.13)	6.4946 ± 8.3616 (12.421, 25.410)
PaCO_2_, kPa (mm Hg)	4.921 ± 0.785 (36.91 ± 5.89)	5.094 ± 0.337 (38.21 ± 2.53)	5.9747 ± 6.1044 (-7.834, 19.784)	5.252 ± 0.332 (39.39 ±2.49)	9.4777 ± 6.8491 (-6.016, 24.971)

*Values in pH, PaO_2_ mm Hg and PaCO_2_ mm Hg have been numerically reported in a previous work [18].

**Figure 1:**
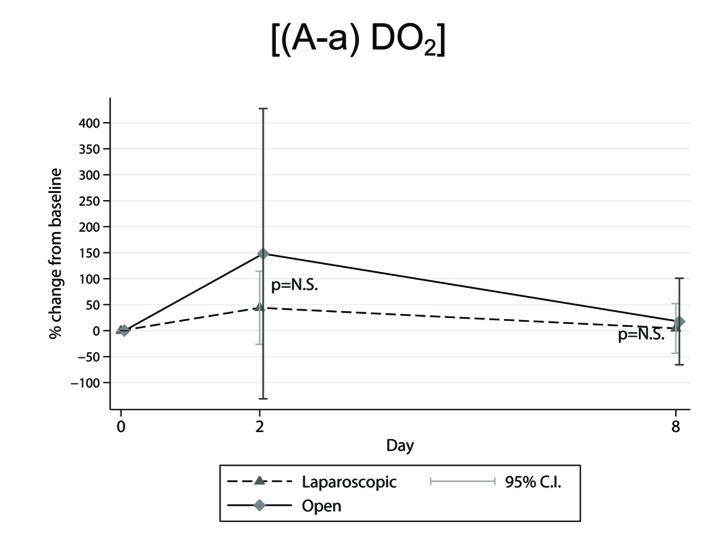
Mean percentage (%) changes in ((A-a)DO_2_) parameter, determined from preoperative level, in the across time measurements between laparoscopic and open cholecystectomy group.

The ((A-a)DO_2_) measurements showed no statistically significant differences between LC and OC patients (Fig. 1), although values 48 h after surgery and percentage (%) changes from preoperative to second postoperative day were detected to be greater in the OC compared to the LC method. Also, from the across time comparisons per technique the overall P-values were found to be at P = 0.06 (tendency for statistical significance) for the LC and P = 0.05 for the OC group. Moreover, the across time measurements per technique showed that for both methods the ((A-a)DO_2_) values were observed to be significantly different between the second and the eighth postoperative days (LC: P = 0.039 vs. OC: P = 0.015).

For the Oct in vol% variable, no statistically significant difference was found between the two groups. However, a reduction from 17.55 ± 1.90 to 15.69 ± 1.88 in the LC group and from 16.99 ± 2.37 to 14.62 ± 2.23 (mean ± SD) in the OC group were shown between the preoperative and the second postoperative day. In addition, a decrease (P = 0.093) for Oct in vol% on the second versus the eighth postoperative day values was also found for the OC group. For the (HCO_3_^-^) variable the across time comparisons either per technique (overall P-values, LC: P = 0.227, OC: P = 0.501) or between the two groups were not significantly different.

Also, the arterial blood oxygen saturation (SaO_2_%) values for either the across time per technique or between the two groups measurements showed no statistically significant differences neither on the second nor on the eighth postoperative day. However, the P-value from the second postoperative day versus baseline between LC and OC method was found at P = 0.096 reflecting a rather greater decrease for the OC compared to the LC patients.

## Discussion

Our work focuses on the investigation of the influence of laparoscopic and open upper abdominal surgery on ABGs-related parameters, particularly on tissue oxygenation, being the ultimate purpose of gas exchange.

The main finding resulting from our experimental study could be summarized as an increase in ((A-a)DO_2_) parameter on the second postoperative day or with the lapse of time after surgery, being more emphatic in the OC compared to the LC group.

By comparing and contrasting the relevant literature with our own findings, the following results were obtained. First, 2 h postoperatively in both LC and OC methods [14] the alveolar-arterial difference in PO_2_ ((A-a)DO_2_) was significantly increased by approximate 45% (P < 0.05) compared to preoperative level. On the first postoperative day the ((A-a)DO_2_) values still remained significantly increased (P < 0.05) compared to baseline in both groups, but for this time period the aforementioned variable was found to be further elevated in the OC resulting in a statistically significant difference (P < 0.05) between the two groups [14]. In our study although our measurements were performed on the second and on the eighth postoperative day, we are in accordance with the previous work [14] in the fact that 2 days after surgery the ((A-a)DO_2_) values were greater in the OC compared to the LC patients. Besides, the evolutionary process between the second and the eighth postoperative day for the recovery of the ((A-a)DO_2_) parameter to preoperative level was more emphatic for the OC group (Fig. 1). Second, no statistically significant differences were observed [21, 22, 24, 25] for bicarbonate concentration values recorded in all the across time measurements. The latter is in accordance with our postoperative findings for this parameter. According to literature, measurements for (HCO_3_^-^) concentrations were made in the operating room in supine position just before the beginning of the operation and during the time of removal of the gallbladder [21], 15 min following induction of anesthesia [24], 1 h after creation of pneumoperitoneum the patient being positioned in the reverse Trendelenburg (10°) with a left tilt (5°) [24], in the supine position just when abdominal’s desufflation was accomplished [24] , in the recovery room 30 min after tracheal extubation [24], and finally from preoperatively to the sixth [22, 25] postoperative day. Third, for SaO_2_% variable a statistically significant decrease was observed on the first postoperative day in both LC and OC methods (the overall P-value at P = 0.0001) without significant difference between groups (P = 0.678) [10]. On the sixth postoperative day SaO_2_% values recovered to their preoperative level in either the LC or the OC patients [10]. In a previous study, Chumillas et al [23] showed that on the second postoperative day SaO_2_% values were significantly reduced (P < 0.003) in the OC compared to the LC method; besides, the postoperative percentage (%) decrease from baseline was significantly greater (P < 0.005) in the OC group [23]. Mimica et al [22, 25] also reported statistically significant decrease in SaO_2_% only in the OC patients. In our study although no statistically significant changes were found, there is an accordance with the aforementioned studies [10, 22, 23, 25] in the fact that the P-value (P = 0.096) for our measurements from the second postoperative day versus baseline between LC and OC reflects a tendency for a greater decrease in the OC group.

Moreover as for the oxygen saturation contribution in evaluation of patients as appropriate candidates for the ambulatory LC, a more recent study [27] showed that the increased patient age was the only statistically significant predictor of lower SaO_2_% levels. Furthermore, smoking habits, male sex and body mass index (BMI) greater than 30 kg/m^2^ were all factors having a part in statistically significant decrease in oxygen saturation values detected at discharge from hospital [27]. Fourth, to our knowledge, Oct in vol% parameter has not been previously reported in literature. A reduction, even without statistically difference between them, was found on the second postoperative day in both LC and OC methods. Fifth, for pH parameter, it has been shown that in patients having undergone LC, values detected during insufflation and in the recovery room 30 min after tracheal extubation were statistically significant decreased (P < 0.05) compared to baseline level [24]. However, in another study, 12 h postoperatively, pH values were significantly reduced (P < 0.02) for patients who underwent OC compared to LC [12]. In another study, pH measurements performed just prior to start of the operation and during the removal of the gallbladder showed no significant changes for the OC and a statistically significant increase (P = 0.006) compared to preoperative for the LC patients [21].

Besides, in a number of studies [10, 11, 15, 17, 22, 25] where pH values were determined preoperatively and in different across time postoperative instants up to the sixth [10, 22, 25] postoperative day, no significant differences were detected in the across time measurements either per technique or between LC and OC surgical techniques.

In our work [18] although postoperative pH changes were minimal for both groups, nevertheless values recorded on the eighth day after surgery showed a statistically significant difference (P = 0.049) between LC and OC in favor of the LC method. Clinical significance could not be attributed to the aforementioned statistical significance due to the fact that the open method constitutes a more invasive technique resulting in more extensive surgical incision and tissue damage. The latter suggestion is supported by another study [28]. Specifically, according to literature [29-31] factors such as increase of intra-abdominal pressure and CO_2_ absorption from the peritoneal cavity both promote the development of acidosis and hypercapnia during the laparoscopic surgical procedure. This is in accordance with Karagulle et al [28] who found that the intra-abdominal pressure resulting from CO_2_ insufflation in laparoscopic surgery bears short-term metabolic, respiratory or mixed-type intraoperative acidosis, being normalized after surgery in the recovery room. Sixth, as for PaO_2_ changes it has been shown [21] that during the operation and specifically at the time of removal of the gallbladder, when arterial blood gases measurements were performed, no statistically significant changes were observed in PaO_2_ levels in both LC and OC. This is in accordance with two studies [12, 16] where no statistically significant differences were displayed for PaO_2_ values, 1 h [16] and 12 h [12] postoperatively in both LC and OC groups. However, 2 h [14] and 6 h [11] postoperatively, PaO_2_ values were found to be significantly reduced (P < 0.05) compared to baseline in the across time measurements per technique in both methods, while statistically significant difference was not detected between LC and OC. Nevertheless, in another two studies, 6 h after the operation [22, 25] the LC-PaO_2_ values remained relatively unchanged compared to their preoperative level whilst in this time period the OC-PaO_2_ values were found to be significantly reduced (P < 0.05 [22] and P = 0.038 [25]), compared to either the LC respective ones or the OC baseline level.

Moreover, on the first postoperative day [10] PaO_2_ was found to be significantly reduced (P = 0.0001) compared to baseline in both LC and OC methods although no statistically significant changes were observed between groups. In five studies [11, 14, 16, 22, 25] 24 h after surgery PaO_2_ values showed statistically significant decrease compared to preoperative level only in patients who underwent OC (P < 0.05) [11, 14, 22]. In addition, on the first postoperative day [14, 16, 22, 25] the OC-PaO_2_ values were also found to be significantly reduced (P < 0.05 [14, 22], P < 0.01 [16], P = 0.011 [25]) compared to LC. In contrast with the previous publications [10, 11, 14, 16, 22, 25], 24 h after surgery no statistically significant changes were detected in both LC and OC and for all measurements [17].

Karayiannakis et al [15] reported that on the second postoperative day the LC-PaO_2_ values returned to their preoperative level, by remaining significantly higher (P < 0.05) compared to the OC-PaO_2_ respective ones. Besides, Chumillas et al [23] found that 48 h after surgery LC-PaO_2_ values were significantly increased (P = 0.002) compared to OC. In the same study [23] on the second postoperative day the OC-PaO_2_ values were also significantly reduced (P < 0.01) compared to their preoperative level. Measurements performed on the third postoperative day showed that PaO_2_ values returned to their preoperative ones in both LC and OC methods, the OC values being lower to LC despite the lack of statistically significant difference between groups [11]. Overall PaO_2_ was significantly higher (P < 0.05 by ANOVA) in the LC compared to the OC method [11].

Furthermore, another study [22] showed that on the third postoperative day the OC-PaO_2_ values remained significantly decreased compared either to their preoperative level or to the respective LC ones (P < 0.05) [22]. In addition to the latter study [22], 3 days after surgery the OC-PaO_2_ values were found to be significantly reduced (P = 0.021) [25] compared to the ones of the LC patients. In a few studies [10, 22, 25] and in both LC and OC surgical procedures, PaO_2_ measurements, either across time per technique or between methods, were also performed on the sixth postoperative day without recording statistically significant difference. In our work [18], PaO_2_ values on the eighth postoperative day had been completely recovered to baseline in both LC and OC groups. However, on the second postoperative day [18], although no significant differences were noted between LC and OC measurements, percentage (%) changes from preoperative to second postoperative day (Table 1, 2) were greater in the OC compared to the LC method. This is compatible with our findings on the second postoperative day for the ((A-a)DO_2_) parameter showing a greater numerical value and percentage (%) change from preoperative to second postoperative day in the OC versus the LC group.

The aforementioned studies [11, 14, 15, 18, 22, 23, 25] focusing on post-LC PaO_2_ values suggest that laparoscopic surgery in the upper abdomen contributes to sustaining better oxygenation. Seventh, in regard with PaCO_2_ parameter, ABGs measurements performed intraoperatively and in the early postoperative period showed that PaCO_2_ values in the operating room and particularly at the time of gallbladder’s removal were significantly increased compared to the ones just prior to surgery only for the LC group of patients [21]. Iwasaka et al [24] also observed that PaCO_2_ values recorded in the recovery room half an hour following tracheal extubation were significantly higher for the LC compared to OC patients. Moreover, 2 h [14] postoperatively PaCO_2_ values were significantly increased (P < 0.05) compared to baseline in both LC and OC methods without statistically significant difference between the two surgical groups. In another study, 6 h after surgery, PaCO_2_ values were significantly decreased (P < 0.05) compared to baseline in both LC and OC patients while significant difference was not detected between methods [22]. However, 12 h [12] postoperatively, the OC group displayed a significantly greater increase (P < 0.03) in PaCO_2_ values compared to LC, suggesting diminished alveolar ventilation in the open surgical method. Moreover, on the first postoperative day, PaCO_2_ values had returned to their preoperative level in the LC group [22]. Twenty-four hours after surgery the OC-PaCO_2_ values remained reduced compared to either their preoperative or to the respective LC ones, having recovered on the third postoperative day [22]. In another work [25] the LC-PaCO_2_ values determined from 6 h following surgery to the sixth postoperative day, remained relatively unchanged compared to their baseline level. As for the OC patients, PaCO_2_ measurements 6 and 24 h postoperatively displayed marginally significant increase (P = 0.056) and decrease (P = 0.044) respectively, compared to laparoscopic group [25].

There are also studies [10, 11, 15-17, 23] where no statistically significant changes were found for PaCO_2_ values in all the across time measurements, either per technique or between methods.

**Figure 2:**
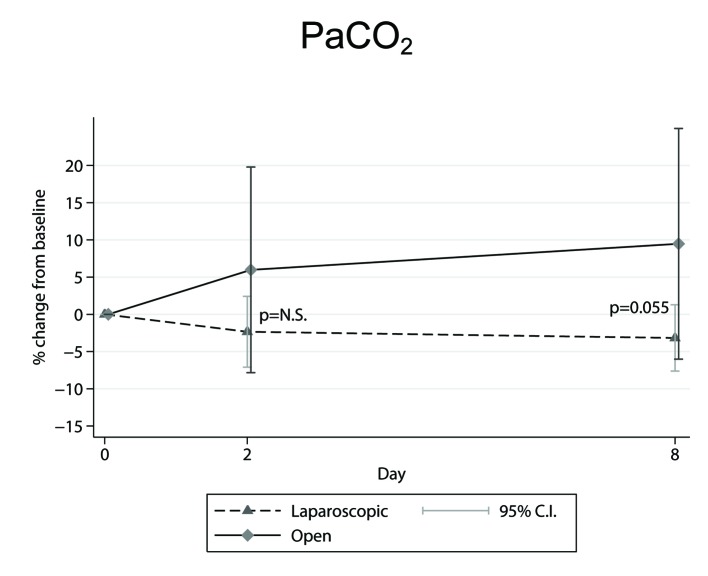
Mean percentage (%) changes in PaCO_2_ parameter, determined from preoperative level, in the across time measurements between laparoscopic and open cholecystectomy group.

In our study [18] the findings for: 1) the percentage (%) changes from preoperative to second and to eighth postoperative day mean ± SEM (95% CI) (Table 1, 2), and 2) the marginally significant increase (P = 0.055) [18] displayed in OC-PaCO_2_ values on the eighth postoperative day compared to the LC ones (Fig. 2), are all indicative of a better oxygenation performance for the laparoscopic surgery.

Focusing on suggestive explanations of our findings regarding all the above studied parameters, the influence of possibly administered analgesics is to be excluded from all our preoperative and postoperative measurements performed either on the second or on the eighth postoperative day. Our post-surgery pH values [18], in both LC and OC groups, were within normal limits and without clinical significance in any postoperative measurement despite the statistically significant difference found on the eighth postoperative day between LC and OC. This absence of clinical significance is well correlated with our finding of bicarbonate concentration stability throughout all measurements before and after surgery.

Also, the tendency for PaCO_2_ to increase during the across time measurements in the OC group [18], may be attributed to the greater increase in breathing frequency, which, according to mean ± SD values on the second postoperative day and the percentage (%) changes from preoperative to second postoperative day mean ± SEM (95% CIs) [20], has been previously shown to be greater in the OC compared to the LC patients [20]. Increased breathing frequency is known to be associated with increased dead space ventilation resulting in PaCO_2_ elevation. Moreover, the decrease in inspiratory capacity that was observed in the OC group [18] seems to have contributed to this effect.

Besides, it is well known that the alveolar-arterial difference in PO_2_ ((A-a)DO_2_) reflects the function of the lung as an oxygenator. The fact that, in our study on the second postoperative day, the ((A-a)DO_2_) variable was found to be greater for the OC compared to the LC group, is postulated to be due to the existence of atelectatic basal alveolar areas that emerge following the more invasive procedure of OC (greater incision, more soft tissue damage, more aggressive surgical manipulations). This has also been supported by Ford et al [32] as a result of the effect of splanchnic and vagal reflexes which inhibit the phrenic nerve during the operation. The above postulation is supported by a previous study [2] according to which abdominal surgery is considered to be a predisposing cause for the appearance of basal-lung areas atelectasis. Also, a recent work [33] showed that during surgery anesthesia introduces disturbances in both alveolar ventilation and perfusion by contributing to atelectasis formation, the latter being unfavorable to arterial blood oxygenation even postoperatively. The explanation was that anesthesia induces loss of muscle tone, bringing about a reduction in either functional residual capacity (FRC) or lung compliance, accompanied by collapsus of lung parenchyma and airway closures [33]. In addition atelectasis produces shunt formation by further deteriorating the oxygenation of arterial blood [33]. Besides, regarding the CO_2_ pneumoperitoneum, although it is in favor of atelectasis production, it does not contribute to shunt formation [33], something which correlates well with our finding of a greater increase in ((A-a)DO_2_) values on the second postoperative day for the OC patients.

OC is undoubtedly associated with more intense postoperative pain [12, 15, 16, 34] which hinders normal sighing being the protective mechanism for prevention of basal atelectasis. We suggest that, according to literature, the resulting atelectatic areas [25] which contribute to ventilation versus perfusion mismatch along with formation of shunt [33], are the main factors of increased ((A-a)DO_2_) difference found in the OC group. The latter is considered to be the cause of a more decreased oxygen saturation which along with the greater blood loss during OC, seems to result in lower blood oxygen content and therefore in a poorer tissue oxygenation for these patients. The explanation of our ((A-a)DO_2_), Oct in vol% and SaO_2_% findings on the second postoperative day for the OC compared to the LC patients, is also probably supported by the fact that for this time period the open surgical procedure was accompanied with greater inflammatory response than the laparoscopic one [12]. The assumption of a greater inflammatory response associated with the open surgical method, is further enhanced by the statistically significant increase found in both erythrocyte sedimentation rate (ESR) 24 h postoperatively (P < 0.02) [12] and C-reactive protein (CRP) values on the first (P < 0.003) [12] and on the second postoperative day (P < 0.04) [12] for the OC compared to the LC patients, taking into account that CRP is considered to be a sensitive indicator of the inflammation [35]. The aforementioned findings [12], especially for CRP, can be explained by Wilmore’s [36] work according to which afferent neural stimuli along with circulating factors are considered to be involved in metabolic responses, such as increased stress hormones release, attributed to surgical trauma. It has also been reported in literature that a single dose of 8 mg of dexamethasone IV results in a significant relief of pain and of stress response after LC [37], the former being further enhanced by using active sub-diaphragmatic aspiration of insufflated CO_2_ [38]. Also, it has been found that oxygenation prior to surgery is considered as a strong predisposing factor in atelectasis appearance [33]. This can be prevented if throughout anesthesia, inflation of the lung is performed by lower airway pressures (40 cm H_2_O) and lower oxygen concentration (less than 40%) [33].

However, in our study the examined oxygenation parameters appear to gradually recover as it is shown by the evolution of their values with the lapse of time. Furthermore it seems that the time needed for complete recovery of ((A-a)DO_2_) parameter between the second and the eighth postoperative day, might probably be critical for patients with deranged lung function in favor of those undergoing LC (Fig. 1).

### Conclusion

Our findings on the second postoperative day for ((A-a)DO_2_) and Oct in vol% parameters showed that the oxygenation status was more affected in patients who underwent the open surgical procedure. This is also supported by the fact that on the eighth postoperative day a marginally significant increase was noted for PaCO_2_ in the OC group as well. We can therefore postulate that LC preserves rather better tissue oxygenation and is presumably more appropriate for patients with chronic lung disease.

## Author Note

The work was conducted in the Pulmonary Function Laboratory of the Intensive Care Unit of the “Korgialeneio-Benakeio” Red Cross General Hospital, Athens, Greece. The protocol of the study was approved by the Department of Experimental Physiology, Medical School, National and Kapodistrian University of Athens, Athens, Greece.
